# Knowledge of obstetric danger signs and birth preparedness practices among women in rural Uganda

**DOI:** 10.1186/1742-4755-8-33

**Published:** 2011-11-16

**Authors:** Jerome K Kabakyenga, Per-Olof Östergren, Eleanor Turyakira, Karen O Pettersson

**Affiliations:** 1 Division of Social Medicine and Global Health, Department of Clinical Sciences, Lund University, CRC, Entrance 72, 205 02 Malmo, Sweden; 2 Department of Community Health, Mbarara University of Science and Technology, P. O. Box 1410, Mbarara, Uganda

## Abstract

**Background:**

Improving knowledge of obstetric danger signs and promoting birth preparedness practices are strategies aimed at enhancing utilization of skilled care in low-income countries. The aim of the study was to explore the association between knowledge of obstetric danger signs and birth preparedness among recently delivered women in south-western Uganda.

**Methods:**

The study included 764 recently delivered women from 112 villages in Mbarara district. Community survey methods were used and 764 recently delivered women from 112 villages in Mbarara district were included in study. Interviewer administered questionnaire were used to collect data. Logistic regression analyses were conducted to explore the relationship between knowledge of key danger signs and birth preparedness.

**Results:**

Fifty two percent of women knew at least one key danger sign during pregnancy, 72% during delivery and 72% during postpartum. Only 19% had knowledge of 3 or more key danger signs during the three periods. Of the four birth preparedness practices; 91% had saved money, 71% had bought birth materials, 61% identified a health professional and 61% identified means of transport. Overall 35% of the respondents were birth prepared. The relationship between knowledge of at least one key danger sign during pregnancy or during postpartum and birth preparedness showed statistical significance which persisted after adjusting for probable confounders (OR 1.8, 95% CI: 1.2-2.6) and (OR 1.9, 95% CI: 1.2-3.0) respectively. Young age and high levels of education had synergistic effect on the relationship between knowledge and birth preparedness. The associations between knowledge of at least one key danger sign during childbirth or knowledge that prolonged labour was a key danger sign and birth preparedness were not statistically significant.

**Conclusions:**

The prevalence of recently delivered women who had knowledge of key danger signs or those who were birth prepared was very low. Since the majority of women attend antenatal care sessions, the quality and methods of delivery of antenatal care education require review so as to improve its effectiveness. Universal primary and secondary education programmes ought to be promoted so as to enhance the impact of knowledge of key danger signs on birth preparedness practices.

## Background

Knowledge of obstetric danger signs and birth preparedness are strategies aimed at enhancing the utilization of skilled care during low-risk births and emergency obstetric care in complicated cases in low income countries [1,2]. The presence of skilled attendants at births and availability of emergency obstetric care have been shown to greatly reduce maternal deaths due to obstetric complications [3-5]. The above-mentioned success, however, depends on a functional referral system from rural communities to health facilities [6]. Facilities with skilled attendants and functional emergency obstetric care services are in most low-income countries located in urban centres whereas the majority of the population live in rural areas. Most maternal deaths in resource poor countries such as Uganda where the actual study was conducted, are attributed to the three delays; delay to make a decision to seek care, delay to reach place of care and delay in receiving appropriate and adequate care [7].

With the assumption that "every pregnancy faces risks" [8,9], women should be made aware of danger signs of obstetric complications during pregnancy, delivery and the postpartum [1,10]. The knowledge will ultimately empower them and their families to make prompt decisions to seek care from skilled birth attendants [11]. Moreover, in order for women to reach the place where appropriate care is provided, certain preparations prior to birth are required. Birth preparedness for a woman entails identifying a skilled attendant/health facility with delivery services, making transportation plans, saving money and identifying a blood donor [1]. The practice of individual women identifying blood donors is, however, discouraged in high HIV/AIDS prevalence countries where voluntary donation to centralised blood banks is preferred [12,13].

Studies conducted among women in Tanzania [11], Ethiopia [14] and Burkina Faso [15] indicate low levels of awareness of obstetric danger signs during pregnancy, delivery and postpartum. Similarly studies have also indicated low rates of birth preparedness among women in Kenya [16], Ethiopia [14,17] and Burkina Faso [15]. The low awareness of danger signs coupled with lack of preparedness contributes to the delay in seeking skilled care henceforth leading to high levels of maternal mortality and morbidity.

With a maternal mortality ratio estimated to range from 215 to 558/100,000 live births [18-20] and with only 42% of women assisted by skilled attendants during birth [20], Uganda is one of the countries still facing the burden of unsafe motherhood. The country target derived from the Millennium Development Goal five (MDG 5) to reduce maternal mortality ratio to 131/100,000 live births may not be achieved unless well-designed and focused interventions are instituted [21]. The government of Uganda has embarked on a road map to accelerate the reduction of maternal/neonatal mortality and morbidity so as to achieve the MDG 5 [22]. One of the strategies laid down in this roadmap is to empower communities to ensure a continuum of care between the household and the health care facility. This will be done by promoting knowledge of danger signs, birth preparedness and complication readiness [22].

The south-western region of Uganda has consistently reported lower rates of women delivering under the care of skilled birth attendants than other regions in the country. The Uganda Demographic and Health Survey (UDHS) report of 2006 showed that skilled attendants assisted only 32% of women in the region which is lower than the national average [20]. Interventions are being designed to accelerate improvement of maternal health through promoting increased skilled attendance at birth in the region. The study was undertaken to explore the association between knowledge of obstetric danger signs and birth preparedness among recently delivered women in Mbarara district of south-western Uganda.

## Methods

### Study design and setting

A community survey of recently delivered women (within the last 12 months) and currently pregnant women was conducted between September 2010 and May 2011 in Mbarara district. Mbarara district is located in the south-western region of Uganda about 270 kilometres from Kampala City. The district covers an area of 1788 square kilometres, has a projected population of 427,200 with 80% working and residing in rural areas. Administratively the district is divided into three counties (health sub-districts); Mbarara municipality, which is the main urban centre while Kashari and Rwampara counties are largely rural with few small towns/trading centres [23]. The majority of the population in the district depends on subsistence agriculture for their livelihood.

The district has 47 health centres of levels II-IV, which among other health services provide antenatal and basic emergency obstetric care services. Mbarara regional referral hospital located in Mbarara municipality is the only public hospital providing comprehensive obstetric care services. The hospital also serves as a referral centre for other general hospitals and health centres within Mbarara and neighbouring districts. The district also is served by four private hospitals all located in Mbarara municipality where two are purely private. The other two are owned by religious organisations and are categorised as private not for profit (PNFP). In Uganda more than 80% of women residing in urban areas deliver under the care of skilled birth attendants compared to 32% of women in rural areas [20].

### Sampling method and Sample size

Two-stage cluster sampling was used to select study participants. In the first stage, a list of villages and the respective number of households was used to independently select similar number of study villages in Kashari and Rwampara counties. In total, 112 out of 699 villages were randomly chosen. In the second stage, women who had either delivered within the previous 12 months or were currently pregnant were randomly chosen in each village with assistance of a Village Health Team (VHT) member. In each village, a starting point was alternately identified at the centre or periphery with the help of a VHT member. Two research assistants moved in opposite direction choosing every other household until 10 women who met the inclusion criteria were interviewed. In total 1199 women recently delivered or currently pregnant were interviewed during the survey. The focus of this article being knowledge of key danger signs and birth preparedness practices, this paper presents the results for the 764 women from the community survey who had delivered within 12 months prior the date of the survey.

### Data collection and management

A safe motherhood questionnaire developed by the Maternal Neonatal Program of JHPIEGO, an affiliate of John Hopkins University [1] was used. It contained four sections namely; socio-demographic information and reproductive history, knowledge on pregnancy and childbirth, experiences related to last pregnancy and childbirth, and exposure to media and interventions. The questionnaire was adapted to fit the Ugandan context and subsequently pretested in the neighbouring district of Isingiro. Some modifications such as including a question regarding purchase of delivery kit/birth materials as a common birth preparedness practice were effected after pretesting. Twelve research assistants (all bachelor's degree graduates of social sciences) with experience in survey data collection were trained for one week, participated in the pretesting and thereafter conducted the interviews under the supervision of Jerome Kabakyenga (JK) and Eleanor Turyakira (ET). Data collection was accomplished in two phases: for Kashari county (September - December 2010) and Rwampara county (April -May 2011). During data collection all questionnaires were checked for completeness and consistency by the field supervisors. All data were coded, and double entered into a database and validated using Epi Data Version 3.1. Data clerks verified all data entry mismatches and made corrections in the database. Further cleaning was done using Stata Version 9 (Stata Corp, Texas).

### Definition of variables

#### Socio demographic variables

County of residence was coded "Kashari" or "Rwampara". Type of residence was categorised as "rural" or "semi-urban". Age was categorised into 2 groups "<25" (young women 16-24), "≥25" (older). Marital status was dichotomised so that "married/in union" was coded "married" and "single", "widowed", "divorced", "separated" was coded "not married". Highest education level completed was dichotomized so that "no formal schooling", "primary" were coded into "<Secondary" and any education beyond primary was coded "≥Secondary". Occupation was dichotomised so that "commercial farmer", "trader", "salaried employment", were coded "regular income" while "house wife", "casual labourer" were coded "irregular income". Religion was dichotomised so that "Roman Catholic", "Church of Uganda", "Seventh Day Adventist" were coded as "Christians" and the rest (Moslems, traditionalists) were coded "others". Household assets ownership (radio, television set, mobile phone, bicycle) were scored (1,2,3,4) so that ownership of two or more assets was classified "high" and ownership of one or none coded "low". Travel from health facility with delivery services was coded "<1 hour" (near) and "≥1 hour" (far)

#### Reproductive variables

Antenatal Care (ANC) attendance - was coded "less than four times" (<4) and "four or more times" (≥4). The variable "Parity" was divided into three groups of "1", "2-4", "≥5".

Key danger signs are those that are common, can easily be recognised and are signs of serious complications [1] and they are grouped under three phases of pregnancy, childbirth and postpartum. The key danger signs during pregnancy include; severe vaginal bleeding, swollen hands/face and blurred vision while key danger signs during childbirths are; severe vaginal bleeding, prolonged labour (labour lasting more than 12 hours), convulsions and retained placenta. The key danger signs during postpartum include; severe vaginal bleeding, foul-smelling vaginal discharge and high fever. The question posed to participants to elicit responses on knowledge of key danger signs during the three phases was "In your opinion, what are some serious health problems that can occur during pregnancy/labour and child birth/in the first 2 days after birth that could endanger the life of a woman?" Only spontaneous responses were recorded.

Knowledge of at least one key danger sign during any of the three phases (pregnancy, childbirth or postpartum) was coded "Yes" or "No".

Birth preparedness: A woman was classified as "well birth prepared" in the most recent pregnancy if she had accomplished three of the following practices: identified skilled health professional, saved money, identified transport or had delivery kit/materials. A woman who made arrangements for birth in less than three of the four ways was classified as "not well birth prepared"

### Statistical analysis

Statistical analysis was performed using Stata Version 9 and all analysis accounted for the intra-cluster correlation. The number and proportion of participants were computed and presented in tables for selected characteristics. Comparisons of the proportion of women who were birth prepared by each category of the independent variables were done and statistical significance assessed using the Chi-square test. Odds ratios and 95% confidence intervals were computed using binary logistic regression. Variables whose association to birth preparedness was statistically significant or if the p-value was less than 0.2 were considered for multivariate analysis. Stepwise multivariable random effects logistic regression with a random intercept was carried out to determine motivating factors for birth preparedness and adjusted for known confounders. The potential effect modification of age, education, and household assets ownership was applied on the association between knowledge of at least one key danger sign during pregnancy or postpartum and birth preparedness was examined using "the departure from additivity criterion" [24].

### Ethical considerations

The Uganda National Council of Science and Technology granted ethical clearance for the study the study. Permission was also sought from local leaders at the district, county and village levels. Interviewers read out the contents of a written consent form to each participant selected for the study. The participant consented by appending a signature or thumbprint on the consent form before the interview commenced.

## Results

Seven hundred and sixty four women, who had delivered within the previous twelve months, were included in the study. Fifty two percent were residents of Kashari while 48% were residents of Rwampara counties (Table 1). The sample age range was 16 to 45 years (mean 27 +/- 6 years) and 40% were young women (16-24 years). The majority were married (95%), Christians (94%), and had lower than secondary education (75%). More than three quarters of the women did not have regular income. However, the majority came from households, which owned mobile phones (63%) or radios (84%). About 60% of the women resided in areas that were located less than one-hour travel time to a health facility offering delivery services. Two thirds of the women had ever been pregnant from 1 to 4 times while nearly a third (32%) had ever been pregnant five or more times.

**Table 1:** Socio-demographic and reproductive characteristics (N = 764)

Characteristics	Number (n)	Percent (%)
**County**		
Kashari	389	51.7
Rwampara	375	48.3
		
**Location of residence**		
Rural	641	77.2
Semi-urban	123	22.8
		
**Age (years)**		
<25	303	39.6
≥25	461	60.4
		
**Marital status**		
Not married	37	4.6
Married	726	95.4
		
**Education level**		
Less than secondary (low)	588	75.3
≥Secondary (high)	175	24.7
		
**Occupation**		
Irregular income	605	76.5
Regular income	157	23.5
		
**Religion**		
Christians	734	94.5
Others	29	5.5
		
**Household assets ownership**		
Low (0-1)	214	27.0
High (*≥*2)	550	73.0
		
**Parity**		
1	163	21.5
2-4	355	46.2
≥5	246	32.3
**ANC Attendance**		
<4 visits	247	32.4
*≥*4 visits	517	67.6
		
**Travel time to health facility**		
<1 hour	418	59.9
≥1 hour	339	40.1

More than two thirds (68%) of the women had attended the minimum recommended four visits of antenatal care and the majority had received education about danger signs (98%), where to go for complications (98%), where to deliver from (98%), identifying a skilled health professional (88%), identifying transport (97%) and saving money (98%). Regarding knowledge of key danger signs, severe vaginal bleeding was the most frequently mentioned complication by women during the following phases; pregnancy (49%), childbirth (64%) and postpartum (57%) (Table 2). Prolonged labour, which is one of the top five major causes of maternal mortality and topmost cause of morbidity in low-income countries, was only reported by 18.3%. The majority of the respondents were able to mention at least one key danger sign in the following phases; during pregnancy (51.8%), childbirth (71.8%) and postpartum (71.6%). However when the scores were combined for the three periods only 18.7% could mention at least 3 key danger signs in all three periods.

**Table 2:** Proportion of women who reported knowledge of key danger signs during pregnancy, childbirth and postpartum (N = 764)

	Knowledge of key danger signs					
	
	Pregnancy		Childbirth		Postpartum	
	
	n	%	n	%	n	%
Severe vaginal bleeding	368	49.2				
Swollen hands/face	58	8.7				
Blurred vision	15	1.6				
Severe vaginal bleeding			484	63.8		
Retained placenta			271	35.1		
Labour lasting more than 12 hours			133	18.3		
Convulsions/fits			16	1.7		
Severe vaginal bleeding					449	56.7
High fever					254	31.0
Foul smelling vaginal discharge					75	9.4

Of the four birth preparedness practices considered in our study; 61% of the respondents had identified a health professional, 91% had saved money and 61% had identified means of transport, while 71% had bought delivery kits/birth materials during their most recent pregnancy. Overall 35% of the respondents were found to have made arrangements in 3 of the four birth preparedness practices and were classified as "well birth prepared".

Table 3 shows the association between selected socio-demographic, reproductive characteristics, knowledge of danger signs and birth preparedness. Women who were from households that had high assets ownership score were more likely to be birth prepared than those with lower household assets ownership score, though this relationship was not statistically significant (OR 1.5, 95% CI: 1.0-2.3). Attendance of antenatal care of four or more times was not associated with being well birth prepared. Women with knowledge of at least one key danger sign during pregnancy or during postpartum were more likely to be birth prepared than those without this knowledge and this relationship was statistically significant with OR 1.9, 95% CI: 1.3-2.7 and OR 2.1, 95% CI: 1.3-3.3 respectively. However the relationship regarding women who had knowledge of at least one key danger sign during childbirth or women who had knowledge that prolonged labour was a key danger sign and birth preparedness was not statistically significant. Table 4 shows stepwise multivariable logistic regression analyses performed to account for age, education and household assets ownership as possible confounders of the association between knowledge of at least one key danger sign during pregnancy or during postpartum as the main exposures and birth preparedness as the outcome. In model one age was adjusted for whereas in model two, education was introduced into the model. In model three household assets ownership score was also added to the model. The association between knowledge of at least one key danger sign during pregnancy (OR 1.8, 95% CI: 1.2-2.6), knowledge of at least one key danger sign during postpartum (OR 1.9, 95% CI: 1.2-3.0) remained statistically significant after adjusting for age, education and household assets ownership as potential confounders.

**Table 3:** Association between socio-demographic, reproductive characteristics, knowledge of key danger signs and birth preparedness

Characteristics	Birth prepared n (%)	OR (95% CI)
**County**		
Kashari	124/382 (31.3)	1.0 (ref)
Rwampara	142/361 (39.4)	1.5 (0.9-2.6)
**Type of residence**		
Rural	237/625 (37.7)	1.0 (ref)
Semi-urban	29/118 (26.5)	0.6 (0.3-1.1)
**Age (years)**		
<25	108/292 (37.4)	0.8 (0.6-1.2)
≥25	158/451 (33.8)	1.0 (ref)
**Marital status**		
Not married	13/36 (37.4)	0.9 (0.4-2.2)
Married	253/707 (35.1)	1.0 (ref)
**Education level**		
Less than secondary(low)	192/571 (32.8)	1.0 (ref)
≥Secondary (high)	74/172 (42.4)	1.5 (1.0-2.3)
**Occupation**		
Irregular income	200/590 (33.5)	1.0 (ref)
Regular income	66/153 (40.8)	1.5 (0.9-2.3)
**Household assets ownership score**		
Low (0-1)	63/207 (31.3)	1.0 (ref)
High (≥2)	203/536 (36.6)	**1.5 (1.0-2.3)**
**Parity**		
1	58/157 (35.2)	0.9 (0.6-1.4)
2-4	130/346 (37.7)	1.0 (ref)
≥5	78/240 (31.6)	0.7 (0.5-1.1)
**Attendance of ANC**		
<4 visits	89/241 (36.7)	1.0 (ref)
≥4 visits	177/502 (34.5)	1.0 (0.7-1.5)
**Travel time from health facility**		
<1 hour	146/411 (35.8)	1.0 (ref)
≥1 hour	120/332 (35.8)	0.9 (0.6-1.3)
**Knowledge of at least 1 key danger sign**		
During pregnancy		
No	104/364 (27.7)	1.0 (ref)
Yes	162/379 (42.2)	**1.9 (1.3-2.7)**
During childbirth		
No	60/210 (27.9)	1.0 (ref)
Yes	206/533 (38.1)	1.5 (0.9-2.3)
During postpartum		
No	51/195 (26.0)	1.0 (ref)
Yes	215/548 (38.9)	**2.1 (1.3-3.3)**
**Knows labour lasting more than 12 hours is a danger sign**		
No	219/612 (34.9)	1.0 (ref)
Yes	47/131 (36.6)	0.8 (0.5-1.4)

**Table 4:** Association (Odds Ratio, 95% CI) between knowledge of at least 1 key danger sign during pregnancy/postpartum and birth preparedness. Multivariable logistic regression

Birth preparedness	Model 1 (Adjusted for Age)	Model 2 (Adjusted for age and education)	Model 3 (Adjusted for age, education and household assets ownership)
			
Factors			
Knowledge of at least 1 key danger sign during pregnancy: *Yes vs. No*	1.8 (1.2-2.6)	1.8 (1.2-2.6)	1.8 (1.3-2.7)
Knowledge of at least 1 key danger sign during postpartum: *Yes vs. No*	1.9 (1.2-3.0)	1.9 (1.2-3.1)	1.9 (1.2-3.1)
Age (years): *≥25 vs. under 25 *	0.8 (0.6-1.2)	0.8 (0.6-1.2)	0.8 (0.6-1.2)
Education: *≥Secondary vs. <Secondary*		1.6 (1.0-2.4)	1.5 (1.0-2.3)
Assets ownership: *high vs. low*			1.5 (1.0-2.3)

Table 5 shows the result of the possible synergistic effect of age, education and household assets ownership on the relation between knowledge of one key danger sign during pregnancy and birth preparedness. A high level of education seemed to have a synergistic effect on the relation between knowledge of key danger signs during pregnancy and birth preparedness. Similarly young age also appeared to have synergistic effect on the association between knowledge of one key danger sign during pregnancy and birth preparedness. However, household assets ownership seemed to have no such synergistic effect on the above relationship.

**Table 5:** Analysis of effect modification between age, education, assets ownership and knowledge of at least one key danger sign during pregnancy regarding birth preparedness presented as adjusted OR with 95% CI

	**Birth preparedness**	
	
**Age/knowledge of 1 key danger sign during pregnancy**	**n (%)**	**OR (95% CI)**
		
Less than 25 years/no knowledge of key danger sign	43 (16.2)	1.0 (ref)
Less than 25 years/had knowledge of key danger sign	65 (24.4)	2.3 (1.3-4.1)
*≥*25 years/no knowledge of key danger sign	61 (22.9)	0.9 (0.5-1.6)
*≥*25 years/had knowledge of key danger sign	97 (36.5)	1.6 (1.0-2.7)
Total	266 (100)	
		
	
Education/knowledge of 1 key danger sign during pregnancy	n (%)	OR (95% CI)
		
Less than secondary/no knowledge of key danger sign	76 (28.6)	1.0 (ref)
Less than secondary/had knowledge of key danger sign	116 (43.6)	1.6 (1.0-2.4)
*≥*Secondary education/no knowledge of key danger sign	28 (10.5)	1.0 (0.5-1.8)
*≥*Secondary education/had knowledge of key danger sign	46 (17.3)	3.7 (2.0-6.8)
Total	266 (100)	
		
	
Household assets ownership/knowledge of 1 key danger sign during pregnancy	n (%)	OR (95% CI)
		
Low assets ownership/no knowledge of key danger sign	20 (7.5)	1.0 (ref)
Low assets ownership/had knowledge of key danger sign	43 (16.2)	2.5 (1.2-5.2)
High assets ownership/no knowledge of key danger sign	84 (31.6)	1.9 (1.0-3.6)
High assets ownership/had knowledge of key danger sign	119 (44.7)	3.4 (1.8-6.5)
Total	266 (100)	

Table 6 shows the result of the possible synergistic effect of age, education and household assets ownership on the relation between knowledge of at least one key danger sign during the postpartum period and birth preparedness. A high level of education seemed to have a clear synergistic effect on the mentioned association. Young age also appeared to have a synergistic effect on the relationship between knowledge of at least one key danger sign during postpartum and birth preparedness. However, high household assets ownership seemed not to have any effect on the relationship between knowledge of key danger signs during postpartum and birth preparedness.

**Table 6:** Analysis of effect modification between age, education, assets ownership and knowledge of at least one key danger sign during postpartum regarding birth preparedness presented as adjusted OR with 95% CI

	**Birth preparedness**	
	
**Age/knowledge of 1 key danger sign during postpartum**	**n (%)**	**OR (95% CI)**
		
Less than 25 years/no knowledge of key danger sign	21 (7.9)	1.0 (ref)
Less than 25 years/had knowledge of key danger sign	87 (32.7)	2.3 (1.1-4.6)
*≥*25 years/no knowledge of key danger sign	30 (11.3)	0.9 (0.4-1.9)
*≥*25 years/had knowledge of key danger sign	128 (48.1)	1.8 (0.9-3.6)
Total	266 (100)	
		
	
Education/knowledge of 1 key danger sign during postpartum	n (%)	OR (95% CI)
		
Less than secondary/no knowledge of key danger sign	37 (13.9)	1.0 (ref)
Less than secondary/had knowledge of key danger sign	155 (58.3)	1.7 (1.0-2.9)
*≥*Secondary education/no knowledge of key danger sign	14 (5.3)	0.9 (0.4-2.0)
*≥*Secondary education/had knowledge of key danger sign	60 (22.6)	3.7 (1.8-6.4)
Total	266 (100)	
		
	
Household assets ownership/knowledge of 1 key danger sign during postpartum	n (%)	OR (95% CI)
		
Low assets ownership/no knowledge of key danger sign	13 (4.9)	1.0 (ref)
Low assets ownership/had knowledge of key danger sign	50 (18.8)	1.7 (0.7-4.0)
High assets ownership/no knowledge of key danger sign	38 (14.3)	2.5 (1.0-5.9)
High assets ownership/had knowledge of key danger sign	165 (62.0)	3.6 (1.6-8.1)
Total	266 (100)	

## Discussion

Our results show a clear association between knowledge of key danger signs during pregnancy or during the postpartum period with birth preparedness among women in rural areas of Mbarara district. The association remained statistically significant even after controlling for possible confounding of age, education and ownership of household assets. A surprising finding in our study was lack of a clear association between knowledge of danger signs during childbirth and birth preparedness. This may be explained by sub-standard health education offered by health care professionals; especially during antenatal care visits. A study conducted in eastern Uganda on the quality of antenatal care provided by midwives found the quality to be poor as less than half of antenatal clinic exit clients interviewed were able to spontaneously recall warning signs of pregnancy complications. About 40% had not been advised where to deliver and that staff were allegedly unfriendly [25]. Another study conducted in the neighbouring district of Rakai [26] reported that most women attended antenatal care to get the ANC card which would grant them access to the health unit or hospital in case of complications. According to our knowledge, there are no published studies conducted in Uganda, which have explored the relationship between knowledge of key danger signs during pregnancy, childbirth and birth preparedness. However studies conducted in Ethiopia [14] and in India [27] found no significant association between key danger signs and birth preparedness after multivariate analyses.

In our study high level of education was found to modify the relationships between knowledge of key danger signs during pregnancy/postpartum and birth preparedness in a synergistic direction. The explanation for this finding could be that women who have attained high levels of education are able to better understand the health messages acquired from various sources. Similarly studies conducted in other countries have separately showed a clear relationship between high education and awareness of danger signs in Tanzania [11] and in Kenya [16]. High levels of education among women have also been associated with increased birth preparedness practices in Ethiopia [14,17] and Kenya [16]. General programmes promoting education of the girl child such as universal secondary education currently being implemented in Uganda [28] would go a long way in promoting better maternal health if safe mother hood is promoted in the syllabus. In our analyses young age was also found to have a possible synergistic effect on the association between knowledge of key danger signs during pregnancy/postpartum and birth preparedness. In support of this observation, the Uganda demographic and health survey 2006 report indicated that young women below the age of twenty years had higher rates of antenatal care attendances and deliveries assisted by skilled birth attendants than older women [20].

The prevalence of birth preparedness of 35% estimated in our study appears to be higher than what was reported from Kenya 7% [16], or 20%- 22% reported in studies from Ethiopia [14,17] but lower than 48%, which was reported by a study conducted in India [27]. It is difficult, however to compare our study findings with those from other as the measures used to determine birth preparedness had some variations and the general environments differed somewhat. Nevertheless, the underlying principles regarding birth preparedness are the same and the methods used to study birth preparedness are similar. The most common birth preparedness practice observed in our study was saving money, which may be explained by the fact that both women and their partners know that money is required to facilitate referral in case of complications. Other studies on birth preparedness in Ethiopia [14] and India [27] have reported similar findings. Studies conducted in Burkina Faso [15] and in Ethiopia [17], however, found that most women had identified skilled birth attendants and health facility as the main birth preparedness practices respectively.

Knowledge of key danger signs is essential for motivating women to seek skilled attendance at birth and also to seek referral in case of complications [11]. In our study the prevalence of knowledge of at least three key danger signs during the three phases; pregnancy, childbirth and postpartum was very low (19%). This may indicate that key danger signs are not emphasised during antenatal care, as our study shows that the majority of the respondents (68%) had attended at least four antenatal care visits during their last pregnancy. Few studies are published on the effectiveness of ANC education however a Cochrane review [29] failed to establish the effectiveness of antenatal education on childbirth and parenthood. Severe vaginal bleeding during pregnancy, childbirth and postpartum was the key danger sign reported by most respondents which may be an indication of awareness by women that bleeding is the main and fastest cause of maternal mortality. However prolonged labour which is a major cause of mortality and debilitating mortality in south-western Uganda [30] was only known by a small proportion of women as a key danger sign in this study. This finding, however, of low knowledge of prolonged labour as a danger sign, is not unique to our study. Studies conducted in eastern Uganda [25], The Gambia [31] and Tanzania [11] have similarly reported that women appear to be unaware of the risk they take by subjecting themselves to prolonged labour in the community.

The finding of high prevalence of mobile phones and radios in households is an opportunity to be exploited by intervention programmes on safe motherhood programmes. Through innovative approaches the mobile phones can be used as channels of providing a continuum of care between families and health care workers. The use of mobile phones has already proved successful in HIV programmes in Uganda [32].

Every woman should be made aware of the likelihood of complications during pregnancy, childbirth/labour and the postpartum periods. Women and their spouses and community members should be availed all the information on danger signs. Our findings indicated low levels of knowledge of danger signs and low levels of birth preparedness (35%) in the rural population studied; however, the same findings would most likely apply to different parts of the country with slight variations. Interventions targeting improvement of maternal health need to consider the quality of antenatal care, including the quality of information offered to pregnant women and their spouses. Knowledge of key danger signs needs to be given priority as it prepares the women and their families for timely and appropriate decision making in case of complications whereas birth preparedness offers readiness to reach health facilities for normal or complicated childbirth.

### Study Limitations

It is possible that there may have been different degrees of recall bias between women who did have complicated pregnancies and those who had uneventful ones. If women with low level of knowledge were more prone to have complicated pregnancies and also better recalled the advices given, this would bias the findings towards the null. However, recall bias could theoretically have worked in the opposite direction as well, i.e. so that the found differences were inflated. Taking this uncertainty into account, we find it unlikely that recall bias distorted our finding to any important degree. There is likelihood that the birthing experience of some women could have modified their responses to questions on knowledge of danger signs or birth preparedness but this could not have adversely affected the findings observed in this study. The explanation is that birth outcome was not an outcome variable in the study. Moreover, it is not feasible to handle the mentioned situation as confounding since it could just as well be a mediating mechanism, since the outcome was determined at the time of the interview. Selection bias was minimised by the random method used to select 112 villages, which were spread out in the various parts of the two counties. The sample of women in our study, most likely represent the population of recently delivered women in rural Mbarara district. Confounding was controlled for in the analysis by stepwise multivariable logistic regression. Possible confounders were introduced into the regression stepwise and they did not have significant effect on the association between knowledge of at least one key danger signs during pregnancy or during postpartum and during birth preparedness.

## Conclusions/Implications

Our study showed low levels of knowledge of obstetric danger signs and low levels of birth preparedness among rural women in south-western Uganda. The study also demonstrated strong association between knowledge of dangers signs during pregnancy, the postpartum period and birth preparedness. The absence of association between knowledge of danger signs during childbirth and birth preparedness is rather surprising. The highest risk of fatal maternal complications is at the time of childbirth and the period just after delivery. Women, spouses and communities need to be empowered with knowledge on obstetric danger signs which are an indication that urgent emergency care needs to be sought from skilled attendants. They also need to be advised to be prepared for child birth through health education using all available channels. The availability of mobile phones and radios need to be utilised by health educators innovatively so as reach as many people as possible. Since the majority of women attend antenatal care clinics district health services and Ministry of Health need to review and improve the quality of antenatal care programmes being delivered. Governmental programmes such as universal primary and secondary education should prioritise safe motherhood programmes in their syllabi. More studies are recommended in the determining the effectiveness of antenatal care education being implemented.

## Abbreviations

ANC: Antenatal Care; HIV: Human Immunodeficiency Virus; MDG: Millennium Development Goal; PNFP: Private not for Profit; UDHS: Uganda Demographic and Health Survey; VHT: Village Health Team.

## Competing interests

The authors declare that they have no competing interests.

## Authors' contributions

JK participated in the study design, data collection and analysis. JK wrote the first draft. P-OO participated in the analysis and reviewed the manuscript. ET participated in data collection and performed statistical analysis. KOP participated in the study design and reviewed the manuscript. All authors read and approved the final manuscript.
